# Silver Nanoparticle Production by the Cyanobacterium *Cyanothece* sp.: De Novo Manipulation of Nano-Biosynthesis by Phytohormones

**DOI:** 10.3390/life12020139

**Published:** 2022-01-18

**Authors:** Maged E. Mohamed, Nermin A. El Semary, Nancy S. Younis

**Affiliations:** 1 Pharmaceutical Sciences Department, College of Clinical Pharmacy, King Faisal University, Al-Ahsa 31982, Saudi Arabia; nyounis@kfu.edu.sa; 2 Pharmacognosy Department, College of Pharmacy, Zagazig University, Zagazig 44519, Egypt; 3 Biological Sciences Department, College of Science, King Faisal University, Al-Ahsa 31982, Saudi Arabia; 4 Botany and Microbiology Department, Faculty of Science, Helwan University, Ain Helwan, Cairo 11975, Egypt

**Keywords:** abscisic acid, GA3, IAA, kinetin, methyl jasmonate

## Abstract

Background: Numerous cyanobacteria have the potential to reduce metallic ions to form pure metal nanoparticles in a green biosynthesis process. Aim: To investigate the production capacity of silver nanoparticles by the cyanobacterium *Cyanothece* sp. and to examine the effect of five different phytohormones, indole acetic acid, kinetin; gibberellic acid; abscisic acid; and methyl jasmonate, on this capacity. Methods: The cyanobacterial strain was grown for 60 days and the harvested cyanobacterium biomass was incubated with 0.1 mM of AgNO_3_. Percentage conversion of Ag^+^ to Ag^0^ was calculated to indicate the AgNPs’ production capacity. Different concentrations of the five phytohormones were added to cultures and the AgNP production was monitored throughout different time intervals. Results: *Cyanothece* sp. biosynthesized spherical AgNPs (diameter range 70 to 140 nm, average diameter 84.37 nm). The addition of indole acetic acid and kinetin provoked the maximum conversion (87.29% and 55.16%, respectively) of Ag^+^ to Ag^0^, exceeding or slightly below that of the control (56%). Gibberellic and abscisic acids failed to elevate the Ag^+^ to Ag^0^ conversion rate (45.23% and 47.95%, respectively) above that of the control. Methyl jasmonate increased the Ag^+^ to Ag^0^ conversion rate to 90.29%, although nearly all the cyanobacterial cultures died at the end. Conclusion: Phytohormones could be used to induce or inhibit the green production of AgNPs with the cyanobacterium *Cyanothece* sp. This novel manipulation technique may have several applications in agriculture or biomedicine.

## 1. Introduction

The unicellular coccoid cyanobacterium *Cyanothece* sp. is an aquatic phototrophic and diazotrophic prokaryotic microorganism. It is capable of performing photosynthesis and fixing carbon dioxide into carbohydrates. Moreover, it fixes nitrogen and converts it into ammonia [1]. Medicinally, the cyanobacterium extract was reported to possess many pharmacological activities such as anti-cancer [2], anti-gastric ulcer activity through acting on *Helicobacter pylori* [3], and anti-myocardial infarction activities [4,5].

Numerous cyanobacteria have the capability to reduce metallic ions or oxides to form pure nanoparticles such as silver, gold, ZnO, and iron nanoparticles [6]. Gold is the most produced nanoparticle from cyanobacteria and there are many examples of this green synthesis process. For instance, the cyanobacterium *Plectonema boryanum* produced octahedral gold nanoparticles in the size range of 10 nm when its cultures were treated with gold chloride solution [7]. Similar conditions were applied to both *Spirulina platensis* and *Calothrix* spp. cyanobacterial cultures to produce spherical gold nanoparticles of 20–30 nm diameters [8,9]. In our previous work, the cyanobacteria *Lyngbya majescula* and *Cyanothece* sp. were able to biosynthesize spherical gold nanoparticles [4,5]. 

Another common metal nanoparticle is silver nanoparticles (AgNPs). These nanoparticles were proven to possess several medicinal and pharmacological activities such as anti-cancer, wound and bone healing, anti-inflammatory, anti-viral, anti-bacterial, and anti-fungal activities [10]. Similar to gold nanoparticle production, cyanobacteria were used as platforms for green synthesis of AgNPs, for example, *Cylindrospermospsis* sp., *Lyngbya* sp., *Synechocystis* sp., and *Synechococcus* sp. strains were all able to produce AgNPs when all cultures with or without the biomass were supplied with 0.1 mM AgNO_3_ solution [11]. 

**Figure 1:**
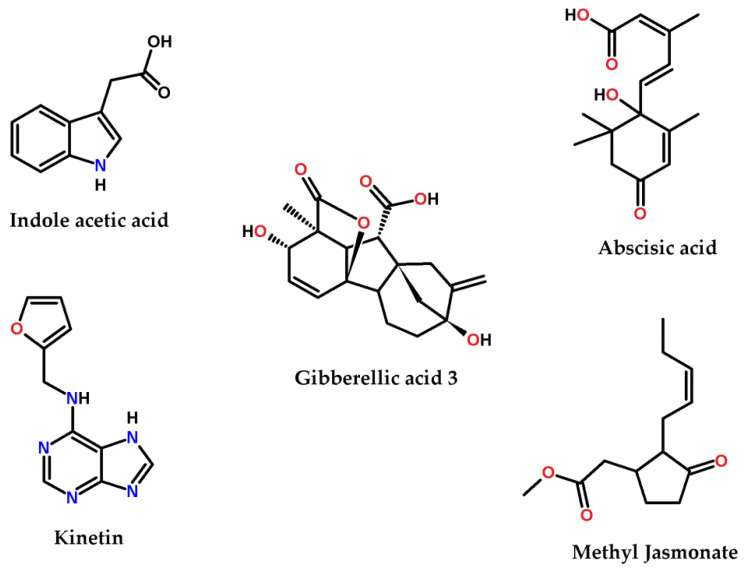
The chemical structure of the phytohormones used in in this study.

Phytohormones are low molecular weight signaling molecules that coordinate many cellular activities in a plant cell. There are many types of these hormones, including auxins, cytokinins, gibberellic acid, ethylene, abscisic acid, salicylic acid, and jasmonic acid. Indole acetic acid (IAA), shown in Figure 1, is the principle auxin in higher plants and has intense influences on plant growth and development and it is one of the most potent physiologically-functioning auxins. Chemically, IAA is a hetero-aromatic organic acid, consisting of an indole ring and an acetic acid side chain. IAA promotes cell elongation in plants, inhibits or delays abscission of leaves, and induces flowering and fruiting [12]. IAA regulates growth and developmental processes such as tissue differentiation, apical dominance, and responses to light, gravity and pathogens. [13] Kinetin, shown in Figure 1, is a 6-aminopurine derivative and is a synthetic plant hormone belonging to the cytokinins. Kinetin stimulates cell division, and therefore controls shoot meristem size and shoot growth. Kinetin accelerates both the differentiation and the outgrowth of axillary buds [14]. Gibberellic acid (GA3), shown in Figure 1, is a plant growth regulator and belongs to the diterpenoid gibberellins. GA3 regulates growth and influences various developmental procedures involving stem germination, elongation, flowering, and enzyme production [15]. Abscisic acid (ABA), shown in Figure 1, is a natural sesquiterpene belonging to the plant growth regulators. ABA hinders growth and metabolism and improves ripening and senescence. ABA has significant functions in seed development and plant stress tolerance due to biotic or abiotic causes [16]. Methyl jasmonate (MG), shown in Figure 1, is a methyl ester which is a member of the volatile compounds in the plant metabolome. MG is a signaling molecule that triggers plant defense against pathogens and herbivores and other biotic and abiotic stresses. MG influences diverse progressive pathways such as seed germination, fruit ripening, and senescence [17]. Cyanobacteria, similar to plant cells, are known to produce some phytohormones such as indole acetic acid (IAA) [18], cytokinins [19], abscisic acid, gibberellins, and ethylene [20]. However, the physiological functions, roles, and effects of such phytohormones in cyanobacteria remain unclear and unidentified [20]. The effect of metal nanoparticles, especially gold and silver nanoparticles, on plant growth and hormonal systems has been extensively studied [21,22,23], however, the other part of the plant hormone/nanoparticle interaction, which is the effect of the plant hormones on nanoparticle production, has, to the best of our knowledge, never been studied. 

Cyanobacteria are easily cultured with minimal costs, which makes them very attractive potential high-throughput producers of green-synthesized AgNPs. The main objective of the present study is to investigate the production capacity of AgNPs by the cyanobacterium *Cyanothece* sp. The effect of different classes and concentrations of phytohormones on the production of the AgNPs was investigated as well.

## 2. Materials and Methods

### 2.1. Isolation and Characterization of the Cyanobacterium Cyanothece sp.

The cyanobacterium was previously collected from marine water samples from Al-Ahsaa, Eastern Province, Kingdom of Saudi Arabia and was kept on F/2 medium [24]. The isolated cyanobacterium has previously been characterized to be *Cyanothece* sp. according to Younis, Bakir, Mohamed, and El Semary [5] using light microscopy.

### 2.2. Production of Silver Nanoparticles in Cyanothece sp. Cultures

AgNP production from *Cyanothece* sp. cultures was implemented according to Hamouda et al. [25] with some modifications. *Cyanothece* sp. culture was grown in F/2 medium for 60 days, and then the cyanobacterial cells were centrifuged (10 min, 2000× *g*). Fifteen micrograms of cyanobacterial biomass was suspended in fresh F/2 medium (19 mL), and 1 mL of 0.1 mM AgNO_3_ was added. The cultures were kept under illumination (2000 ± 200 lux) and rotation (150 RPM) for 72 h. A negative control was prepared through repeating the above process and replacing cyanobacterial biomass or AgNO_3_ with distilled water. Changes in the external solution color to yellow and then brown indicated the production of AgNPs. Two milliliters were taken at zero, 12, 24, 48, 72 h to calculate the percentage conversion of Ag^+^ to Ag^0^ as mentioned below and this 2 mL was replaced with a fresh solution of the medium containing the same concentration of AgNO_3_. 

### 2.3. Characterization of the AgNPs Produced by the Cyanobacterium Cyanothece sp. Cultures

The shape and size of the AgNPs produced were investigated through the dynamic light scattering technique using a Zetasizer Nano (Malvern Instruments Ltd., Worcestershire, UK). Further investigation of nanoparticle shape and size was performed using transmission electron microscopy techniques (TEM, a JEOL JEM-1230, Tokyo, Japan), operated at an accelerating voltage of 200 kV. Samples were prepared by the addition of a drop of the AgNP suspension onto a carbon-covered copper grid and it was then dried under an infrared lamp prior to the examination. To determine the nanoparticle size distribution, digitized TEM images (30 randomly chosen fields) were processed with Image Pro-Plus software (Media Cybernetics, Rockville, MD, USA) and the data obtained were reported in a histogram.

AgNPs were characterized using a UV–Vis spectrometry scan (Genesys10S, Thermo Scientific, Madison, WI, USA) in the range of 200–900 nm. A Fourier transform infrared spectrometer (FT-IR spectrometer, Agilent Cory 630, Agilent Technologies, (Bruker Alpha, Billerica, MA, USA)) was used to detect the functional groups involved in the bioproduction of AgNPs through an investigation region of 4000–500 cm^−1^.

### 2.4. Determination of Percentage Conversion of Ag^+^ to Ag^0^

The percentage of conversion of Ag^+^ to Ag^0^ was used to express the system capacity for nanoparticle production. This percentage was determined according to Rahman et al. [26]. The 2 mL sample (see above) was centrifuged (5 min, 1000× *g*) and the supernatant was recovered. The supernatant was divided into two equal portions (test and control). An equal molar concentration of NaCl (to the original AgNO_3_ concentration) was added to the test sample, while the same amount of distilled water was added to the control sample. NaCl was added to precipitate all unconverted Ag^+^ as AgCl, while it did not affect the AgNP formation or dispersion [26]. The two samples were left for 12 h and then centrifuged (10 min, 1000× *g*) and 50 μL of concentrated HNO_3_ was added to the supernatant (to give a final concentration of 5% HNO_3_). The solution was vortexed and then analyzed to determine silver concentration using inductively coupled plasma–optical emission spectrometry (ICP-OES, Optima 8000, Perkin Elmer Waltham, MA, USA). In the control sample, the whole concentration of added silver was determined, however, in the test sample, only the concentration of Ag^+^ converted to Ag^0^ was determined (as all free Ag^+^ ions were precipitated using NaCl). The percentage conversion was calculated as follows:

C% = (Silver concentration in test/Silver concentration in control) × 100(1)

where C% is the percentage conversion of Ag^+^ to Ag^0^.

### 2.5. Examination of Different Phytohormones’ Effect on AgNP Production

Different concentrations of the phytohormones (indole acetic acid (IAA; 50, 100, 500, 1, and 2 mM final concentration), kinetin (1, 3, 5, 7, and 10 mM final concentration), gibberellic acid (GA3 10, 20, 40, 80, 100 μM final concentration), abscisic acid (1, 3, 5, 7, 10 μM final concentration), and methyl jasmonate (5, 10, 20, 50, and 100 μM final concentration)) were added to the 15 mg cyanobacterium solution in 19 mL of the fresh F2 medium mentioned above prior to the addition of 1 mL of 0.1 mM AgNO_3_. The cultures were incubated as mentioned above for 72 h and then the percentage conversion of Ag^+^ to Ag^0^ was identified as mentioned above at the same time intervals. 

### 2.6. Statistical Analysis

Data generated by the experiments were analyzed by applying response surface methodology (RSM) using Design-Expert version 12.0 software (Stat-Ease, USA), considering concentration of the phytohormones and time as factors and the percentage conversion of Ag^+^ to Ag^0^ as a response using the central composite design method. Data were statistically analyzed using analysis of variance (ANOVA) and data were made into model graphs, such as a 3D response surface plot. The best model to fit all responses was the quadratic model and the p-value for all responses was less than 0.0001. Other statistical analyses were performed using GraphPad software (version 5, San Diego, CA, USA), especially to produce the 2D correlation. Each experiment was repeated 5 times (n = 5). Data are presented as mean ± standard deviation (SD).

## 3. Results

### 3.1. Production of AgNPs by Cyanothece sp. Cultures

**Figure 2:**
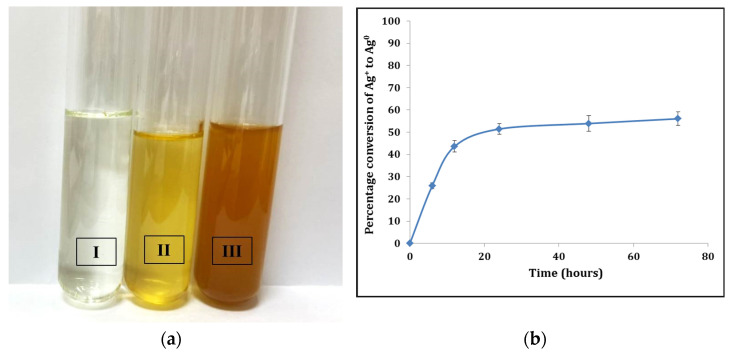
Production of AgNPs from *Cyanothece* sp. cultures. (**a**) The yellow color indicates the biosynthesis of AgNPs in the bacterium culture (II, III) in comparison with the control (I, bacterium culture medium without Ag^+^ source). (**b**) Percentage conversion of Ag^+^ to Ag^0^ metal nanoparticles in 72 h interval. Percentage conversion was calculated according to the methodology in Section 2.4.

When *Cyanothece* sp. cultures were supplied with silver ions as AgNO_3_, the cyanobacteria reacted with the metal ions and converted it into nanoparticles. This conversion could be visually identified through the gradual increase in yellow color intensity, sometimes reaching a pale orange to brown color (Figure 2a). The cyanobacterium culture was able to convert 56% of the Ag^+^ to nanoparticles after 72 h (Figure 2b).

### 3.2. Particle Size and Shape of AgNPs Produced by the Cyanobacterium Cyanothece sp. Cultures

**Figure 3:**
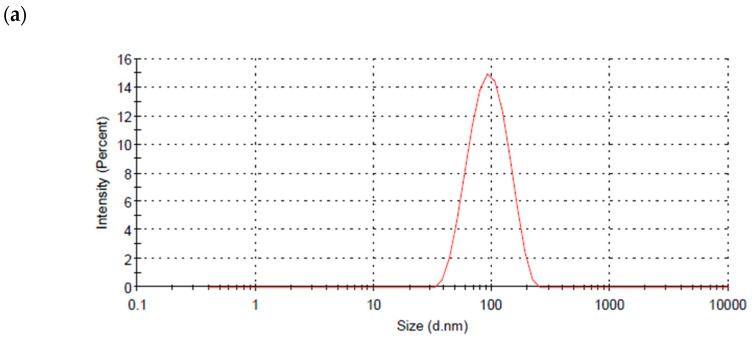

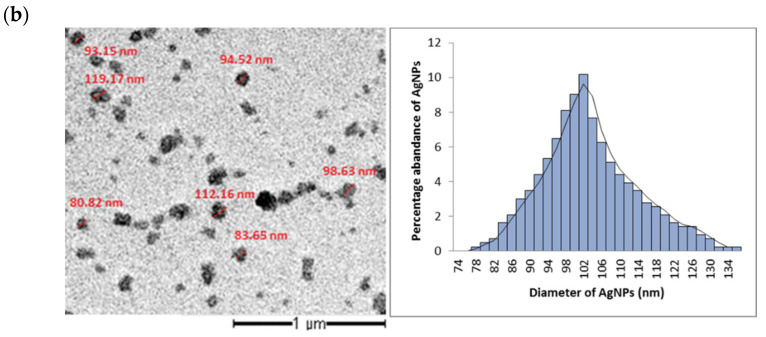
Detection of shape and size of AgNPs produced by the cyanobacterium *Cyanothece* sp. cultures. (**a**) Dynamic scattering graph identifying the particle size, (**b**) TEM images of the AgNPs with measured diameter (in red) and histogram of size distribution.

The dynamic light scattering technique was used to measure the particle size of the produced AgNPs, which was 75–135 nm (Figure 3a). TEM images showed that particles were spherical in shape with a diameter ranging from 70 to 140 nm, with the average particle size being 84.37 nm. The most abundant nanoparticles are those of a diameter of 98 nm (Figure 3b).

### 3.3. Characterization of the AgNPs Produced by the Cyanobacterium Cyanothece sp. Cultures

**Figure 4:**
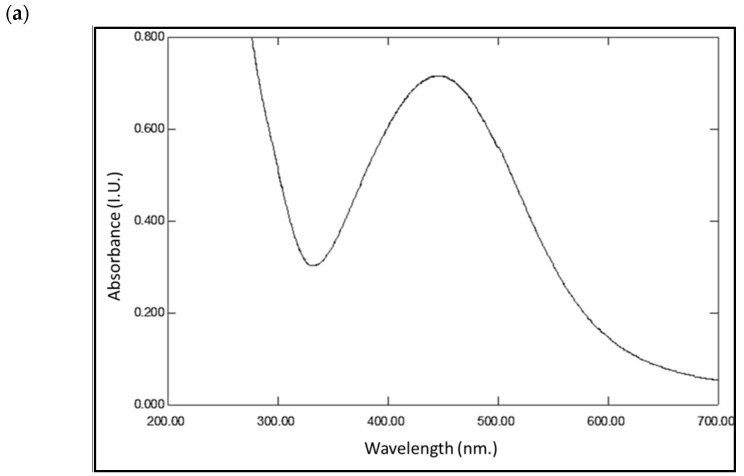

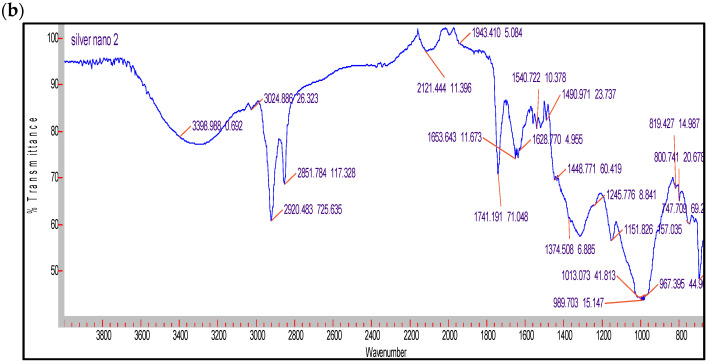
Characterization of the AgNPs produced by the cyanobacterium *Cyanothece* sp. cultures. (**a**) UV–visible light spectrometry. (**b**) FTIR spectrum of dry biosample of cyanobacterium biomass 72 h after AgNO_3_ addition.

The UV–Vis spectrum of the external solution showed surface plasmon resonance within 410–480 nm, with a characteristic peak at 450 nm which is characteristic of nanosilver [27] (Figure 4a). The FTIR of the biosample containing air-dried silver nanoparticles within the biomass, shown in Figure 4b, revealed the presence of several aromatic, alkane, amide, or amine function groups [28]. For example, at 3398 cm^−1^ (O–H stretch, H–bonded alcohols, phenols), 2920 cm^−1^ (C-CH3 stretch, alkanes), 2851 cm^−1^ (CH2, alkanes), 2121 cm^−1^ (C≡C stretch, alkynes), 1653 cm^−1^ and 1628 cm^−1^ (N–H bend, primary amines), 1490 cm^−1^ (C–C stretch (in-ring), aromatics), 1151 cm^−1^ (C–N stretch, aliphatic amines), 1013 cm^−1^ (C–O stretch, alcohols, ethers), 967 cm^−1^ (C-H out of plane bending, aromatics), 819 cm^−1^ (C-H out of plane bending, aromatics), 747 cm^−1^ (C–Cl stretch, alkyl halides). 

### 3.4. Examination of Different Phytohormones’ Effects on AgNP Production

**Figure 5:**
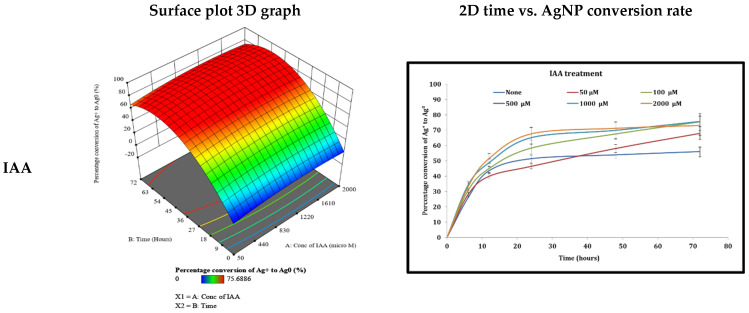

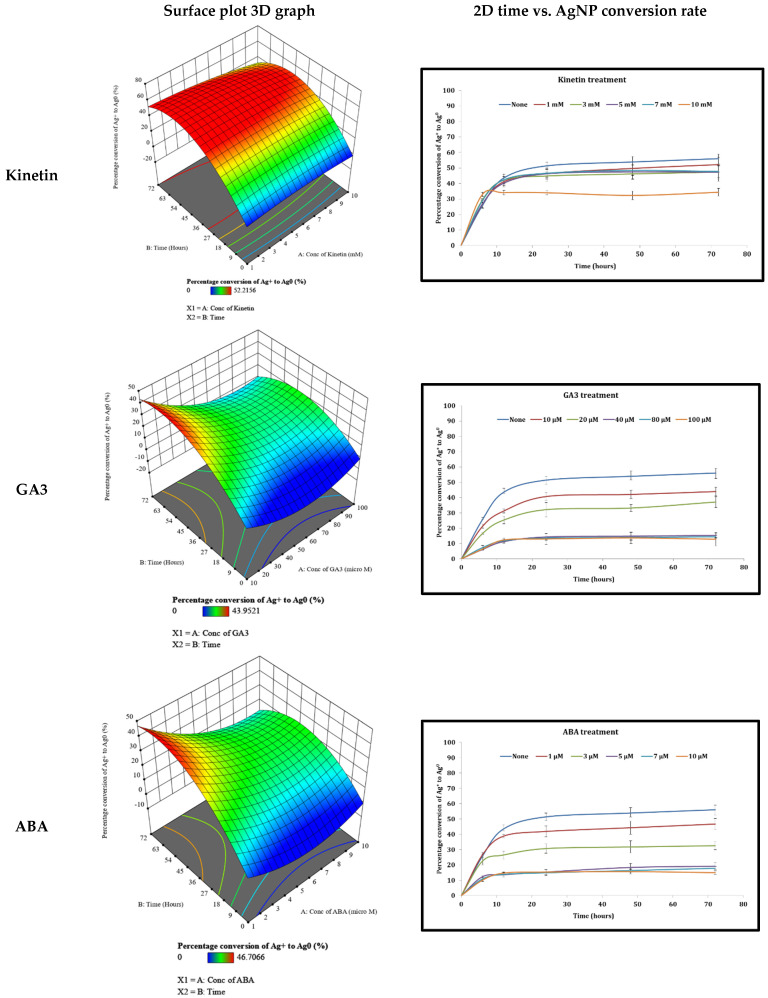

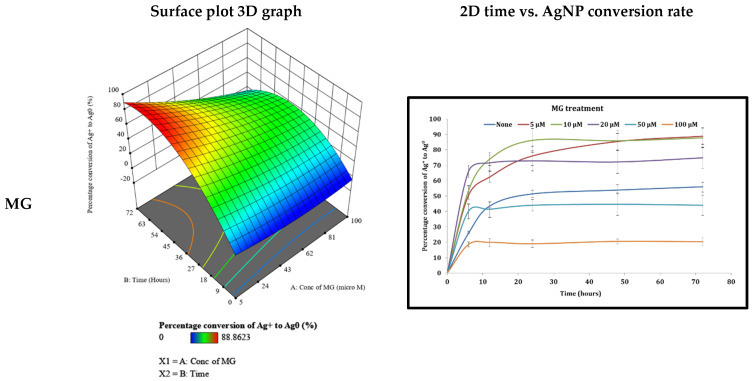
The effect of different phytohormones on the ability of the cyanobacterium *Cyanothece* sp. to produce AgNPs. The left panel represents the surface plot 3D graph (two factors: phytohormone concentration and time versus the response; the percentage conversion of Ag^+^ to Ag^0^). The right panel represents 2D time versus the percentage conversion of Ag^+^ to Ag^0^ for 5 different concentrations of the phytohormone. The phytohormones used are: indole acetic acid (IAA) (50, 100, 500, 1, and 2 mM final concentration), kinetin (Kinetin) (1, 3, 5, 7, and 10 mM final concentration), gibberellic acid (GA3) (10, 20, 40, 80, 100 μM final concentration), abscisic acid (ABA) (1, 3, 5, 7, 10 μM final concentration), methyl jasmonate (MG) (5, 10, 20, 50, and 100 μM final concentration).

The effect of different phytohormones with various concentrations on the production of AgNPs was investigated. The two-dimensional time against the percentage conversion of Ag^+^ to Ag^0^ (Figure 5) graph for all IAA concentrations indicated an increase in the percentage conversion of Ag^+^ to Ag^0^ with time and concentration. The surface plot analysis, shown in Figure 5, indicated that the increase in time and IAA concentration resulted in an intensification in the percentage conversion of Ag^+^ to Ag^0^ to a maximum of 87.29% at the concentration of 1172.26 μM after 45.24 h (Table 1). All the cyanobacterial cultures were green in color after the addition of IAA at all concentrations, however, after 72 h with the 1000 and 2000 μM IAA concentrations, the bacteria started to become yellowish in color, indicating stressful conditions (Table 2).

Several concentrations of kinetin (1, 3, 5, 7, and 10 mM) were investigated for their effect to induce AgNP production within 72 h. All kinetin concentrations gave nearly a similar response to the control (no phytohormones used), as shown in Figure 5, except for the 10 mM kinetin which caused a significantly smaller response. The surface plot analysis (Figure 5) showed that the increase in time but not kinetin concentration resulted in an upsurge in the percentage conversion of Ag^+^ to Ag^0^, reaching a maximum of 55.16% at the concentration of 7.514 mM and after 40.90 h (Table 1). All the cyanobacterial cultures were green in color after the addition of kinetin in all concentrations, however, after 72, 24, and 12 h with the 5, 7, and 10 mM concentrations, respectively, the cyanobacteria started to become yellowish in color, signifying stressful conditions (Table 2).

Gibberellic acid (GA3, 10, 20, 40, 80, 100 μM) gave lower responses than the control, as shown in Figure 5. The surface plot analysis (Figure 5) showed neither time nor concentration escalation significantly increased the Ag^+^ to Ag^0^ conversion rate, which accomplished a maximum of 45.23% at the concentration of 10.56 μM and after 61.42 h (Table 1). All the cyanobacterial cultures started to be under stress (turned yellow in color) after 48 and 24 h with the 80 and 100 μM concentrations, respectively (Table 2). 

Abscisic acid (ABA, 1, 3, 5, 7, 10 μM) caused the cyanobacteria to convert Ag^+^ to Ag^0^ at a significantly rate lower than the non-phytohormone-treated control, as shown in Figure 5. The increase in time or ABA concentration did not significantly elevate the rate of conversion of Ag^+^ to Ag^0^ (maximum rate was 47.95% at the concentration of 1.065 μM and after 52.81 h, Table 1) as indicated in the surface plot graph in Figure 5. All the cyanobacterial cultures started to be under stress (turn yellow in color) after 72, 48, and 24 h with the 5, 7, and 10 μM concentrations, respectively (Table 2). 

Methyl jasmonate (MG, 5, 10, 20, 50, and 100 μM) showed a stress pattern where the first three concentrations gave responses better than the control, reaching a nearly 90% Ag^+^ to Ag^0^ conversion rate; however, the last two concentrations used caused a significantly reduced response relative to the control, shown in Figure 5. The surface plot analysis, shown in Figure 5, indicates a maximum Ag^+^ to Ag^0^ conversion rate of 90.29% with 8.581 μM of MG and after 54.12 h (Table 1). Many of the cyanobacterial cultures suffered death several hours after the addition of MG, as shown in Table 2. 

## 4. Discussion

### 4.1. Prodcution of AgNPs from Cyanothece sp. Cultures 

*Cyanothece sp*. is a unicellular cyanobacterium which is able generate carbohydrates through the use of daylight and to fix atmospheric nitrogen in the soil as ammonia. *Cyanothece sp*. has recently demonstrated the ability to biosynthesize different sizes of gold nanoparticles when treated with gold ion solution [5]. This proven ability has encouraged the authors to investigate the bacterium’s ability to detoxify silver ions to silver nanoparticles (AgNPs). In the current study, cyanobacterium has proven its ability to reduce silver ions in metallic silver and to develop metallic nanoparticles. The particle size of the biosynthesized AgNPs ranged from 70 to 140 nm, as confirmed by dynamic light scattering and TEM analyses. The UV–visible spectrum analysis displayed a surface plasmon resonance peak within 370–410 nm, indicating the presence of AgNPs [27]. The FT-IR spectrum analysis indicated the presence of cellular biomolecules which may be responsible for the stabilization of the biosynthesized silver nanoparticles. Molecules such as aromatics, alkanes, amides, or amines may function as the capping ligand in the formation of silver nanoparticles and also perform the stabilization of silver nanoparticles in an aqueous medium [28]. *Cyanothece* sp. has an uncomplicated metabolomics matrix, comprising mostly polysaccharides, polyphosphates, pigments, and fatty acids. *Cyanothece* sp. produces many polysaccharides, such as exopolysaccharides [29], homoglucan [30], and sulfated polysaccharides [31]. Furthermore, the cyanobacterium produces different types of fatty acids including those of the C18 n-9 and n-3 types [32]. All these metabolites could be the source of the function groups identified by the FT-IR and could be used for the stabilization of the AgNPs produced. 

### 4.2. The Effect of Different Phytohormones on AgNPs’ Production from Cyanothece sp. Cultures

Phytohormones, including auxins, cytokinins, gibberellic acid, abscisic acid, and jasmonic acid-related compounds, manage, organize, and harmonize many growth and metabolite-related activities in plant cells and similar organisms. Many phytohormones were detected in cyanobacteria, including indole acetic acid (IAA) [18], cytokinins [19], abscisic acid, gibberellins, and ethylene, but with unclear functions [20]. The influence of metal nanoparticles on plant growth and hormonal systems has been extensively studied before [21,22,23]. However, the effect of the plant hormones on nanoparticle production via cyanobacteria has hardly been investigated and this study could contribute to this obscure area of scientific knowledge. In this study, the effect of five phytohormones, at different concentrations and time intervals (factors), on the percentage conversion of Ag^+^ to Ag^0^ (response) was explored. The data generated by the experiments were analyzed through the formation of a 16-experiment matrix for each phytohormone using the central composite design method. 

The IAA model revealed an increase in the percentage conversion of Ag^+^ to Ag^0^ with time and concentration to a maximum of 87.29% at the concentration of 1172.26 μM and after 45.24 h. IAA is an essential mediator in plants which operates not only as a plant growth factor but also as a principle substance for stress tolerance [33]. Moreover, it lessens heavy metal damage such as Cu-induced stress damage [34]. For instance, 50 μmol/L IAA could increase Cu metal challenge ability in wheat plants and oppose its induced damage. Ouzounidou and Ilias [35] showed that a 100 µmol/L IAA application diminished the lethal effects of Cu metal in sunflower roots and promoted root growth hair development [36]. Therefore, it could be the stress tolerance effect of IAA which helped the cyanobacteria to continue producing the AgNPs while the phytohormone concentration increased for up to 72 h. This could be supported by the healthy status of the cyanobacterial cultures, which remained green in color nearly all through the experiment (Table 2). 

Externally supplied phytohormones amend the harmful effect of heavy metal on green algae. For example, phytohormone homeostasis was disturbed by lead as a heavy metal, while exogenous auxin and cytokinin application improved this adverse effect [37]. The addition of external kinetin to the cyanobacterial cultures in this study resulted in an increase in the percentage conversion of Ag^+^ to Ag^0^ which reached a maximum of 55.16% at the concentration of 7.514 mM and after 40.90 h. Khalil et al. [38] demonstrated that the treatment of *Phaseolus vulgaris* plants with salicylic acid alone or in combination with kinetin increased the plants’ resistance to nickel and/or lead stress through enhanced antioxidant systems, proline accumulation, and reduced reactive oxygen species, and also stabilized membrane stability [38]. The cytokinin zeatine increases resistance to heavy metals in the halophyte plant species *Kosteletzkya pentacarpos* [39]. These studies indicate that kinetin could allow the cyanobacterium to produce silver nanoparticles as a mechanism to control the toxicity of heavy metals, which is supported by the results of our study. 

Gibberellins or gibberellic acids consist of a family of diterpenoids, an important group of phytohormones that have diverse influences on the growth and development of plants, such as germination, expansion of leaves, development of flowers, and cell elongation. In the present study, the external addition of gibberellic acid to the cyanobacterial cultures did not increase the rate of conversion of Ag^+^ to Ag^0^ to more than 45.23% at the concentration of 10.56 μM and after 61.42 h, which is lower than that produced by the control (56% conversion of Ag^+^ to Ag^0^ after 72 h). Several studies disclosed that gibberellic acid relieves various abiotic stresses, including heavy metal toxicity. Gibberellic acid ameliorated Cd toxicity in *A. thaliana*, by reducing Cd uptake and lipid peroxidation [39]. Exogenous addition of GA3 elevated daidzein and genistein contents in soybean under stress conditions, suggesting the protective effect of GA3 [40]. Siddiqui et al. [41] illustrated GA3’s ameliorative effect against Ni-induced toxic effects in wheat seedlings. Additionally, Gangwar et al. [42] mentioned the protective effect of exogenous additions of gibberellic acid against the toxic effects of Cr in pea seedlings by regulating oxidative stress and the antioxidant system. Moreover, El-Monem et al. [43] have reported that gibberellic acid alleviates the damaging effects of Cd and Pb on broad bean and lupin plants by regulating activities of proteases, CAT, and POD. These studies distinctly suggest the role of gibberellic acid in defending plants against heavy metal stress; however, the presence of gibberellic acid in high concentrations or with prolonged contact could have affected the growth of the cyanobacterium (see Table 2) which could have resulted in the decrease in the percentage of Ag^+^ to Ag^0^ conversion. 

Abscisic acid (ABA) is a natural sesquiterpene phytohormone with essential roles in plants, as it controls plant growth and development, dormancy, senescence, and abscission, with a fundamental role in plant responses to stresses [44]. In our study, increased ABA concentrations or contact time did not significantly increase the rate of conversion of Ag^+^ to Ag^0^, which reached a maximum of 47.95% at the concentration of 1.065 μM and after 52.81 h. Previous investigations suggested that ABA has an important function in abiotic stress response and signal transduction when resisting adverse environments, and plays a principle role in the mitigation of heavy metal stresses in plants [45,46]. Many studies state that ABA drives plant resistance to toxic metals such as As, Cd, and Pb [47,48]. The application of low concentrations of ABA decreased Cd uptake through inhibiting transcription of IRT1 and therefore diminished Cd-induced growth inhibition [48]. However, our study demonstrated that that ABA inhibited the cyanobacterial cells from producing AgNPs and the pattern of the 3D surface plot suggested that the increase in the phytohormone concentration or increase in the length of contact resulted in decreasing the ability to convert Ag^+^ to Ag^0^. The reason behind this behavior needs more investigations, although it can be explained, in part, as a factor of the cyanobacterial culture’s stress and survival (Table 2). 

Methyl jasmonate (MG) is a significant signaling molecule, well recognized for its functions during plant growth, defense, and stress responses [49]. MG is a strong elicitor that affects an enormous variety of biochemical and physiological processes. In our study, MG showed a strange pattern of activity, as it induced Ag^+^ to Ag^0^ conversion responses better than the control, which nearly reached a maximum of 90.29% when a concentration of 8.581 μM of MG was used and after 54.12 h. However, all the cyanobacterial cultures showed symptoms of stress and many of these cultures died before the end of the experiment. MG has a good activity in resisting heavy metal toxicity in plants. Treatment with a low concentration of MG significantly diminished the translocation and accumulation of the toxic Cd metal in *Solanum nigrum* [50], *Glycine max* [51], and wheat seedlings [52]. However, MG is a well-known plant stress hormone, which causes activation of programmed cell death and defense mechanisms in plants. MG induces reactive oxygen species (ROS) production, causing a series of alterations in plant mitochondrial dynamics, including the termination of mitochondrial movement and the loss of mitochondrial transmembrane potential [49]. Therefore, the pattern of MG activity could be explained as the compound causing the cyanobacteria to be stressed, allowing the increase in Ag^+^ to Ag^0^ conversion in less time, followed by the death of the cyanobacterial cells, which hindered the further production of AgNPs.

## 5. Conclusions

The cyanobacterium *Cyanothece* sp. was able to biosynthesize spherical AgNPs within the diameter range of 70 to 140 nm, with the average diameter being 84.37 nm. The most abundant particle diameter was 98 nm. The nanoparticles produced could have been biosynthesized and stabilized by many function groups of different compounds secreted by the bacterium. The effect of five different phytohormones on the ability of the cyanobacterium to produce AgNPs was investigated, giving three different patterns of activity; IAA and kinetin succeeded in inducing the production of AgNPs to a maximum of 87.29% and 55.16%, respectively, exceeding or similar to that of the control (56% conversion rate in 72 h). The second pattern was achieved by GA3 and ABA, which failed to elevate the Ag^+^ to Ag^0^ conversion rate (45.23% and 47.95%, respectively) above that of the control and caused slight stress to the cyanobacterial cells. The third pattern was for MG, which succeeded in elevating the Ag^+^ to Ag^0^ conversion rate to 90.29%, although the compound caused the cyanobacterial cells to die at the end. Therefore, this study recommends the use of IAA to induce the biosynthesis of AgNPs by the cyanobacterium *Cyanothece* sp. MG can be used for a period less than 6 h, and the cyanobacterium’s viability should be monitored throughout the experiment. This study’s results could encourage investigating if phytohormones enhance the properties of the cyanobacterium to detoxify heavy metals as well as the ability of the bacterium in the green production of AgNPs for pharmaceutical and industrial purposes. 

## Figures and Tables

**Table 1:** Ideal predicted values of the factors (concentration of the phytohormone and time) to obtain the maximum response (conversion of Ag^+^ to Ag^0^) for each of the phytohormones according to the surface plot analysis (see Figure 5).

Phytohormone *	Ideal Predicted Factors for Maximum Response		Maximum Response
	Concentration	Time (Hours)	Conversion of Ag^+^ to Ag^0^ (%)
**IAA**	1172.26 (μM)	45.24	87.29
**Kinetin**	7.514 (mM)	40.90	55.16
**GA3**	10.56 (μM)	61.42	45.23
**ABA**	1.065 (μM)	52.81	47.95
**MG**	8.581 (μM)	54.12	90.29

* Indole acetic acid (IAA), kinetin (Kinetin), gibberellic acid (GA3), abscisic acid (ABA), methyl jasmonate (MG).

**Table 2:** The behavior of the cyanobacteria *Cyanothece* sp. in response to the addition of phytohormones over time. The green highlight means that the bacteria are still viable; the yellow highlight means the bacteria are becoming yellow in color and dying, the red highlight means total bacterial death (the cyanobacteria were identified under a light microscope).

Phytohormone *	Concentration	Behavior of Cyanobacteria over Time					
		0 (h)	6 (h)	12 (h)	24 (h)	48 (h)	72 (h)
**None**	None						
**IAA**	50 (μM)						
	100 (μM)						
	500 (μM)						
	1000 (μM)						
	2000 (μM)						
**Kinetin**	1 (mM)						
	3 (mM)						
	5 (mM)						
	7 (mM)						
	10 (mM)						
**GA3**	10 (μM)						
	20 (μM)						
	40 (μM)						
	80 (μM)						
	100 (μM)						
**ABA**	1 (μM)						
	3 (μM)						
	5 (μM)						
	7 (μM)						
	10 (μM)						
**MG**	5 (μM)						
	10 (μM)						
	20 (μM)						
	50 (μM)						
	100 (μM)						

* Indole acetic acid (IAA), kinetin (Kinetin), gibberellic acid (GA3), abscisic acid (ABA), methyl jasmonate (MG).

## Data Availability

No support data.
